# Epitalon protects against post-ovulatory aging-related damage of mouse oocytes *in vitro*

**DOI:** 10.18632/aging.204007

**Published:** 2022-04-12

**Authors:** Xue Yue, Sai-Li Liu, Jia-Ni Guo, Tie-Gang Meng, Xin-Ran Zhang, Hong-Xia Li, Chun-Ying Song, Zhen-Bo Wang, Heide Schatten, Qing-Yuan Sun, Xing-Ping Guo

**Affiliations:** 1 Department of Biochemistry and Molecular Biology, Basic Medical College, Shanxi Medical University, Taiyuan 030001, Shanxi, China; 2 Shanxi Bethune Hospital, Shanxi Academy of Medical Sciences, Reproductive Science Institute, Taiyuan 030032, Shanxi, China; 3 State Key Laboratory of Stem Cell and Reproductive Biology, Institute of Zoology, Chinese Academy of Sciences, Beijing 100101, China; 4 Department of Veterinary Pathobiology, University of Missouri, Columbia, MO 65211, USA; 5 Fertility Preservation Lab, Guangdong-Hong Kong Metabolism and Reproduction Joint Laboratory, Reproductive Medicine Center, Guangdong Second Provincial General Hospital, Guangzhou 510320, Guangdong, China

**Keywords:** aging *in vitro*, oocyte, antioxidant, Epitalon, mitochondria

## Abstract

The developmental potential of oocytes decreases with time after ovulation *in vivo* or *in vitro*. Epitalon is a synthetic short peptide made of four amino acids (alanine, glutamic acid, aspartic acid, and glycine), based on a natural peptide called epithalamion extracted from the pineal gland. It is a potent antioxidant, comparable to melatonin, that may confer longevity benefits. The current study aims to test the protective effects of Epitalon on the quality of post-ovulatory aging oocytes. Epitalon at 0.1mM was added to the culture medium, and the quality of oocytes was evaluated at 6h, 12h, and 24h of culture. We found that 0.1mM Epitalon reduced intracellular reactive oxygen species. Epitalon treatment significantly decreased frequency of spindle defects and abnormal distribution of cortical granules during aging for 12h and 24h, while increased mitochondrial membrane potential and DNA copy number of mitochondria, thus decreasing apoptosis of oocytes by 24h of *in vitro* aging. Our results suggest that Epitalon can delay the aging process of oocytes *in vitro* via modulating mitochondrial activity and ROS levels.

## INTRODUCTION

Healthy oocytes are essential for successful fertilization and subsequent embryo development [1]. Ovulated oocytes are blocked for a prolonged time in the second meiotic metaphase until fertilization takes place [2]. During this period, if not timely fertilized, oocytes will enter into a senescence state *in vivo* and *in vitro*. To increase the success rate of assisted reproductive technology (ART), it is necessary to find effective methods to prevent or delay the aging of oocytes *in vitro* [3].

Aging of oocytes directly affects fertilization and embryo developmental ability, resulting in embryo malformation and pregnancy failure [4]. Aging oocytes show a lower fertilization rate [5], or a higher polyspermy rate [6, 7]. They are characterized by abnormal spindle arrangement, decreased chromosomal integrity [4, 6, 8, 9], disordered cortical granule discharge [9, 10], sclerosis of zona pellucida, and increased sensitivity to parthenogenetic activation [6, 9]. Furthermore, mitochondrial activity of aging oocytes is reduced and dysfunctional [6, 11]. In addition, mitochondria are the main production sites of reactive oxygen free radicals [12]. When the function of mitochondria declines, the ability to remove reactive oxygen species (ROS) decreases, resulting in a decrease of mitochondrial membrane potential (MMP) and DNA copy number of mitochondria (mtDNA copy number) mutations, thus causing cell apoptosis [13, 14].

In order to reduce the age-related damage of oocytes caused by prolonged culture *in vitro*, it is necessary to find an agent that has no negative effect on oocyte development and can effectively delay the aging damage caused by the accumulation of ROS. At present, studies mainly explored effective measures to delay the aging of oocytes from the aspects of antioxidants [15], regulation of MPF activity [16, 17], regulation of oocyte metabolism [18], and regulation of apoptosis signaling pathways [19]. Epitalon was made based on the amino acid analysis of epithalamin (concentrate of the pineal gland-a neuroendocrine system bioregulator, which is the sum of peptides) and has the following sequence of amino-acids: Ala-Glu-Asp-Gly [20]. This short peptide is known for more than 30 years already. Initially, the substance was found in the brain extracts of animals and tested on animals. In male rats, Epithalamin not only produces a direct antioxidant effect but also stimulates the expression of SOD, ceruloplasmin, and other antioxidant enzymes [21–23]. *In vivo*, the research data have proved that Epitalon has a regenerating and life-prolonging effect, as well as a positive effect on the hormonal levels, immune responses [24] and thyroid gland [25]. *In vitro*, Epitalon helps to prevent the early aging process of cells, and inhibit tumor development in somatic cells. In this study, we investigated whether Epitalon prevents post-ovulatory oocyte aging *in vitro*.

## RESULTS

### Epitalon reduces the level of reactive oxygen species in aged oocyte

**Figure 1:**
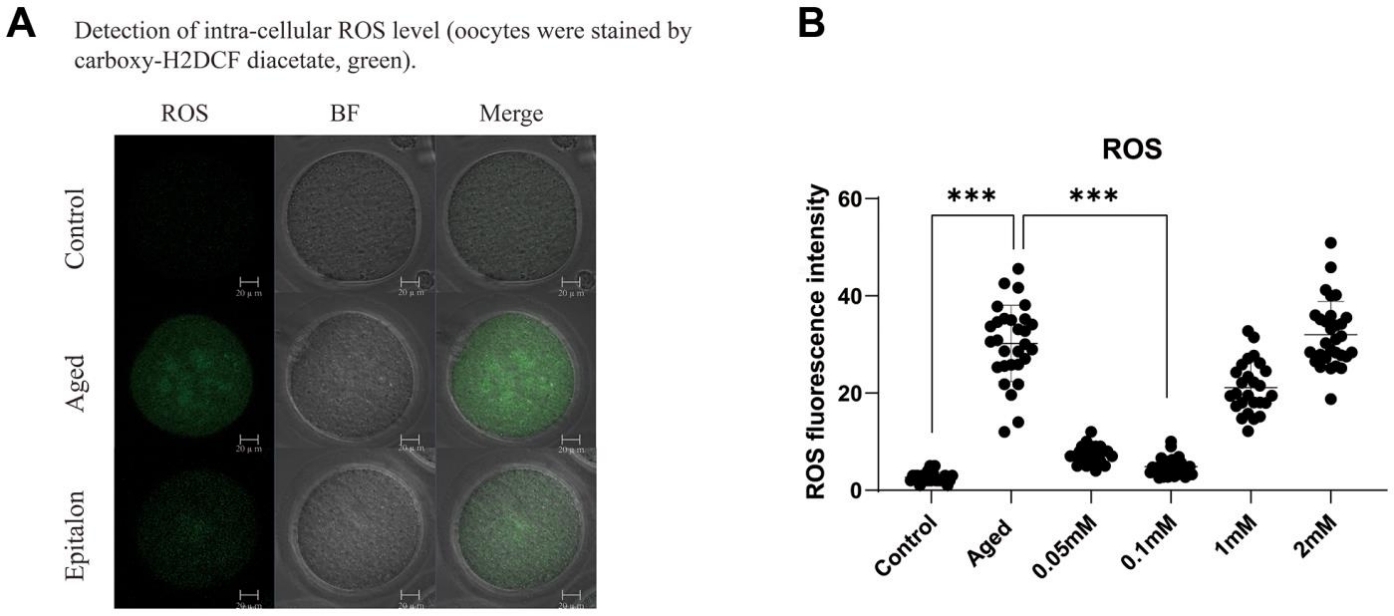
**Epitalon decreased the intracellular ROS level.** (**A**) Representative images of ROS fluorescence of in the Control, Aged, and 0.1mM Epitalon-treated aging oocytes. Images were analyzed by Confocal microscopy with identical fluorescence parameters. (**B**) Quantitative analysis of ROS fluorescence intensity in Control, Aged, 0.05mM, 0.1mM, 1mM, 2mM Epitalon groups. The fluorescence intensity analysis for each oocyte was conducted using Image J software. Data from more than 30 oocytes were analyzed for each group. Significant difference between the Aged and 0.1mM Epitalon-treatment groups was observed (p < 0.01). Data are expressed as mean ± SEM of at least three independent experiments. Scale bar: 20 μm.

A previous study showed that the accumulation of intracellular ROS is an important manifestation of post-ovulatory oocyte aging. Moreover, ROS accumulation was more obvious after 24h *in vitro* culture, so we chose oocyte aging for 24 hours to detect the efficiency of Epitalon scavenging ROS *in vitro*. We first chose to add 0.05mM, 0.1 mM, 1 mM, or 2 mM Epitalon in M2 medium and analyzed the ROS contents in oocytes aging *in vitro* for 24 hours. We found that when the oocytes were exposed to 0.1mM Epitalon, the level of ROS was significantly reduced compared to the Aged group, while 1mM and 2mM Epitalon unexpectedly could not rescue the ROS accumulation in the aged oocytes (Figure 1). Based on the above analysis, we concluded that 0.1mM concentration of Epitalon could reduce ROS in aging oocytes.

### Epitalon decreases the frequency of fragmentation in the post-ovulatory aged oocytes and during parthenogenetic activation

**Figure 2:**
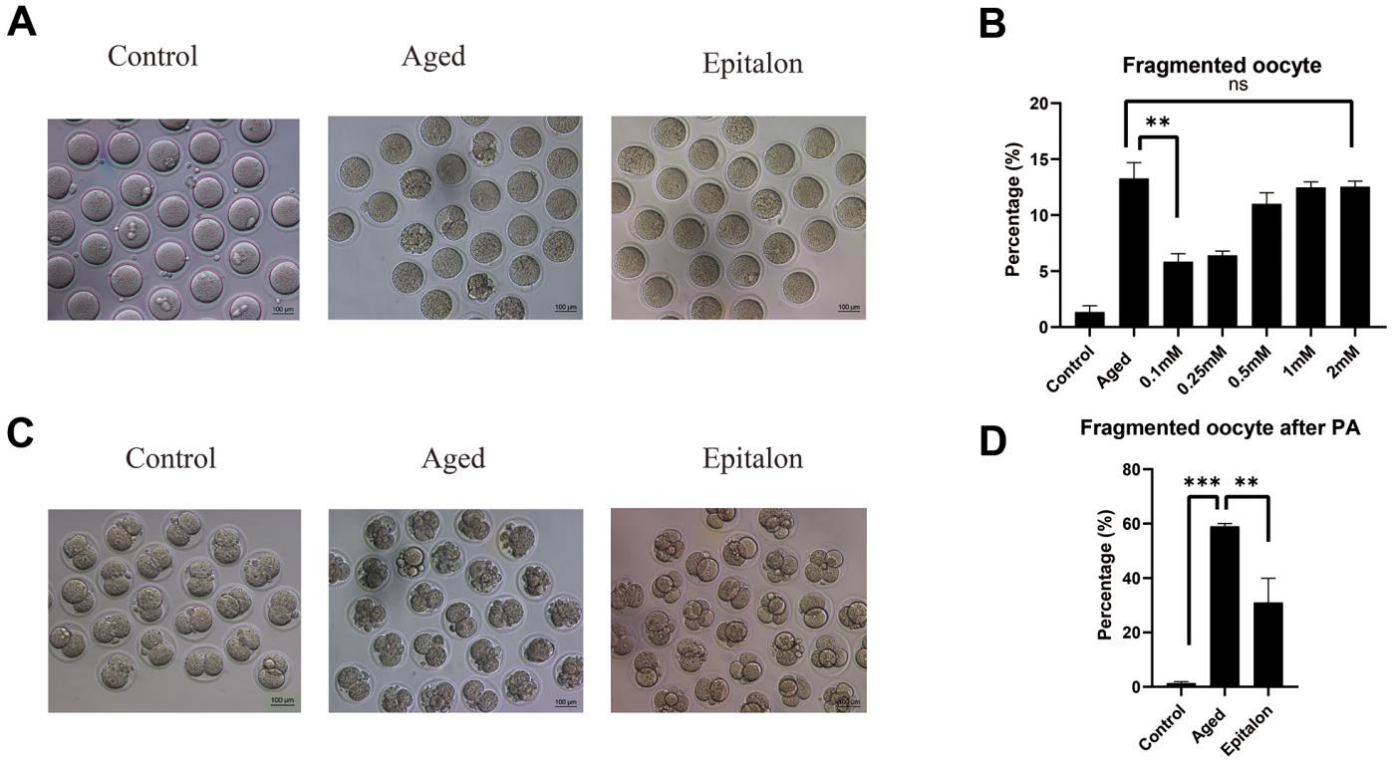
**Effects of Epitalon on the integrity of postovulatory aged oocytes and after parthenogenetic activation.** (**A**) Representative images of fragmented oocytes in the Control, Aged and 0.1mM Epitalon-treated groups. (**B**) The rates of oocyte fragmentation were recorded in the Control, Aged and Epitalon-treated oocytes. Epitalon was supplemented to the culture medium with a concentration of 0.1mM, 0.25mM, 0.5mM, 1mM, or 2mM. (**C**) Representative images of 2-cell embryos from the Control, Aged and 0.1mM Epitalon-treated groups. (**D**) The rates of fragmentation after parthenogenetic activation were recorded in the Control, Aged, and 0.1mM Epitalon-treated oocytes. Data from more than 30 oocytes were analyzed for each group. Significant difference between the Aged and 0.1mM Epitalon groups was observed (p < 0.01). Data are expressed as mean ± SEM of at least three independent experiments. Scale bar: 100 μm. PA, parthenogenetic activation.

The most intuitive way to measure the quality of oocytes is to observe whether oocytes can maintain the integrity of the cytoplasm during aging. Firstly, we detected the morphology and integrity of oocytes aged 24 h *in vitro* after Epitalon supplementation and counted the incidence of fragmentation. As shown in Figure 2, most control fresh oocytes were normal in morphology. However, fragmentation occurred more frequently in the aged group (Figure 2A). To determine the protective effect of Epitalon on the integrity of aging oocytes, ovulated oocytes were cultured with increased dose of Epitalon (0.05, 0.1, 0.25, 0.5, 1, 2mM) for 24 h. Strikingly, 0.1mM Epitalon significantly reduced the fragmentation rate, from 13% in the aged group to 5.8% in the Epitalon supplement group (Figure 2B). We also measured the rate of oocyte fragmentation during parthenogenetic activation of aged oocytes in three groups. Results showed that the Epitalon group reduced the rate of cytoplasmic fragmentation in parthenogenetic activation by approximately 27.0% (Figure 2C, 2D). These results suggest that Epitalon can protect integrity of aged oocytes.

### Epitalon restores the spindle integrity and rescues mislocalized cortical granules

**Figure 3:**
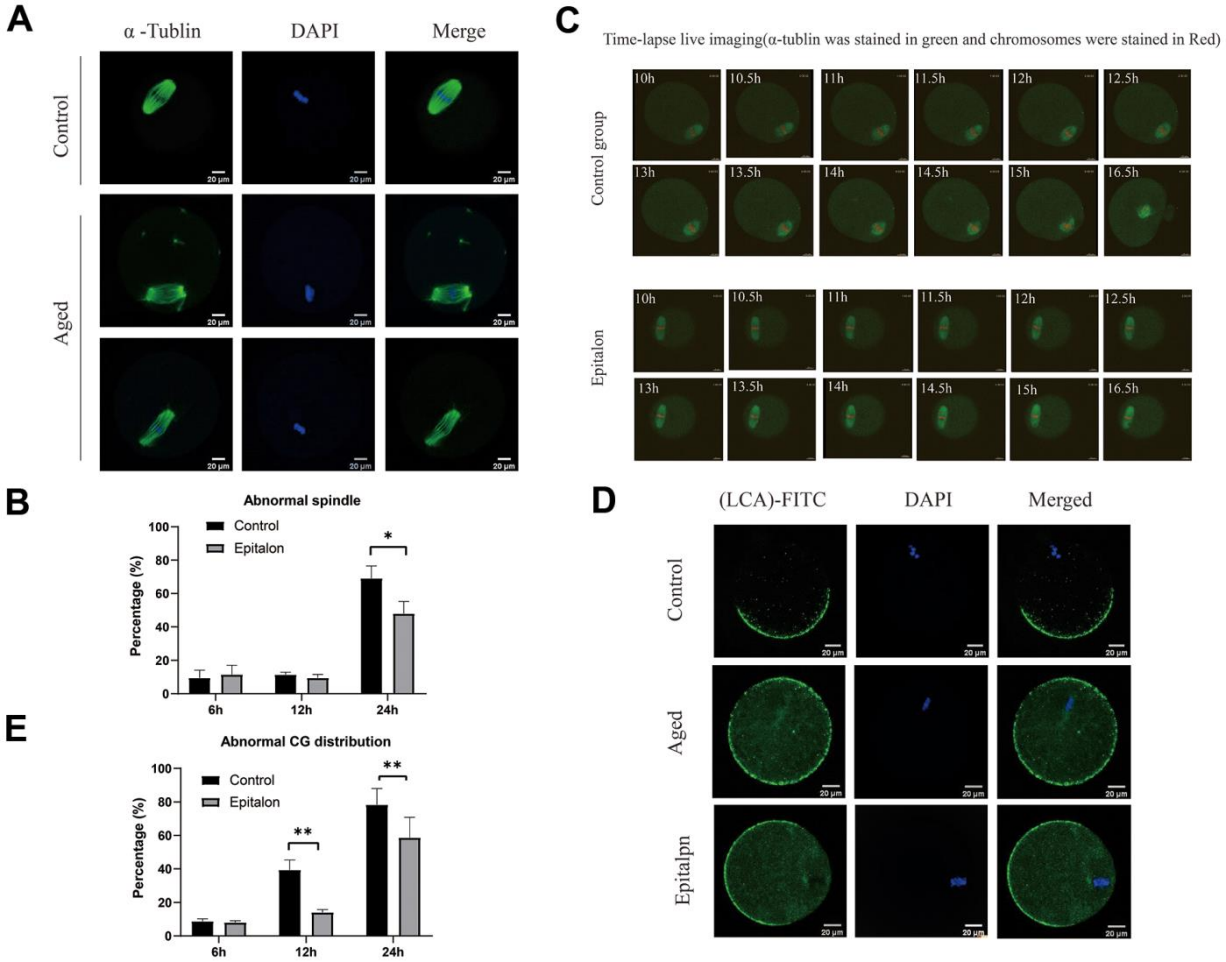
**Epitalon maintained normal spindle integrity and CGs distribution.** (**A**) Different morphological structures of spindles appeared in the Control and aged oocytes. Meiotic spindles in oocytes were stained with α-tubulin (green) and chromosomes were stained with Hoechst 33342 (blue). (**B**) Percentages of abnormal spindles in oocytes. Oocytes containing normal spindles and those with abnormal spindles were counted to calculate the percentage of abnormal spindles. (**C**) Dynamic spindle and chromosome changes in the Control and Epitalon treated oocytes, which were microinjected with MAP4-eGFP mRNA and H2B-mCherry mRNA, and visualized by time-lapse live-cell imaging. The spindles were marked in green, and chromosomes were marked in red. Independent replicates were conducted with a minimum of 20 oocytes. (**D**) Confocal laser scanning microscopy for the distributions of cortical granules (CGs) of oocytes. Metaphase II oocytes were immunolabeled for CGs with lens culinaris (LCA)-FITC (fluorescein isothiocyanate: green), and chromosomes were counterstained with DAPI (blue). (**E**) Percentages of abnormal distribution of CGs in oocytes. Significant difference between the Aged and 0.1mM Epitalon-treated groups was observed (p < 0.05). Data are expressed as mean ± SEM of at least three independent experiments. Scale bar: 20 μm.

It is well known that aging of oocytes causes numerous morphological and cellular changes, including alterations and displacement of spindle structure, misalignment of chromosomes, displacement of first polar body and CGs, and premature exocytosis of CGs. In order to test whether Epitalon has a protective effect on spindle integrity in oocytes, we added 0.1mM Epitalon to the culture medium to observe spindle integrity at 6h, 12h, and 24h of culture. We found that the degree of spindle abnormalities was low before 12h, but increased significantly at 24h, and the spindle defects could be partially rescued by Epitalon (Figure 3A, 3B), suggesting that Epitalon can reduce oocyte spindle defects. The protective effect of Epitalon on spindle integrity was also confirmed by time-lapse live imaging (Figure 3C).

The distribution of CGs is usually considered to be another important indicator of oocyte cytoplasmic quality. We evaluated whether Epitalon could protect CGs distribution pattern by staining with its marker LCA-FITC. Confocal microscopy showed that an atypical distribution pattern of CGs appeared in post-ovulatory oocytes aging *in vitro*. Oocytes displayed a layer of CGs beneath the oolemma and an evident CG-free domain in the area of the MII spindle apparatus were regarded to have normal CGs distribution. During post-ovulatory oocyte aging, CG congregation occurred and moved to CG-free domains near the chromosomes (Figure 3D). The percentage of abnormal distribution of CGs increased gradually with the extension of culture time *in vitro*, and the degree of abnormal distribution increased significantly at 24h. However, compared with aging oocytes, the oocytes treated with 0.1mM Epitalon showed a lower percentage of abnormal CG distribution at 12h and 24h (Figure 3E), indicating its protective effect on CGs distribution of post-ovulatory oocyte *in vitro*.

### Epitalon rescues mitochondrial membrane potential and mtDNA copy number during post-ovulatory oocyte aging *in vitro*

**Figure 4:**
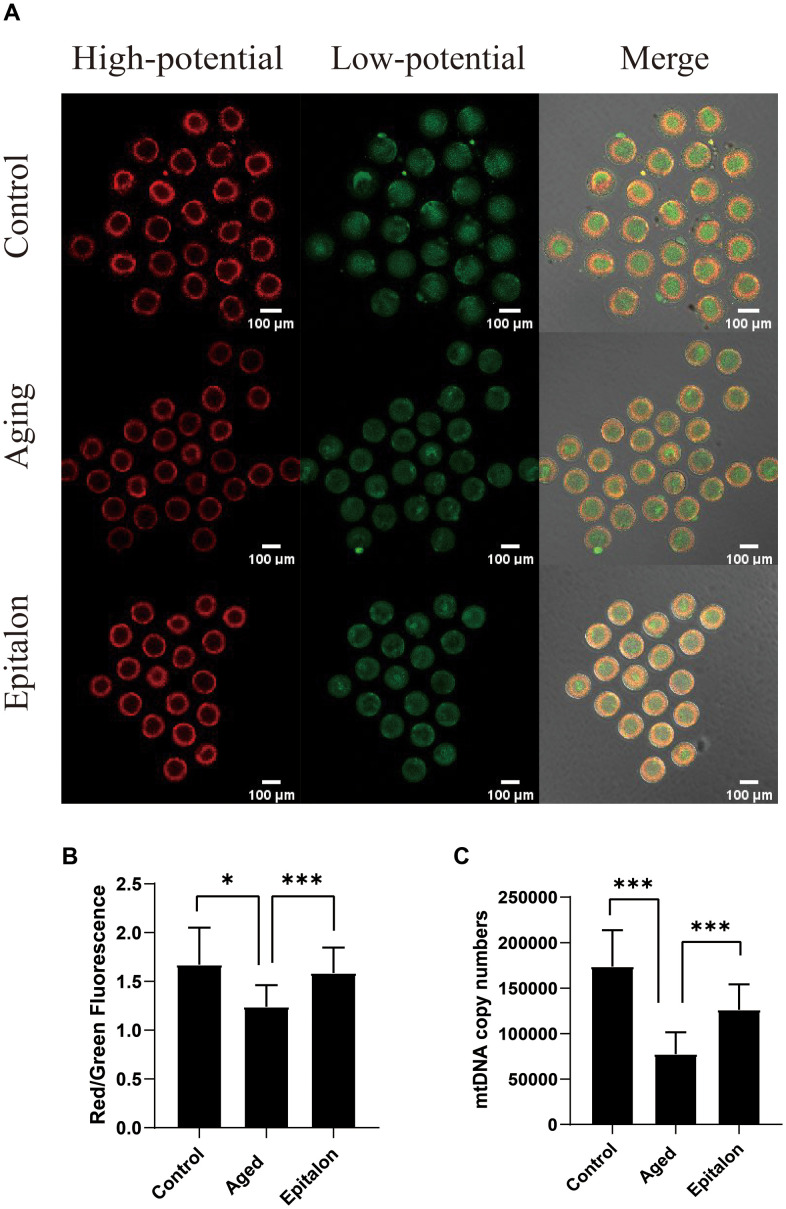
**Epitalon protected the function of mitochondria during post-ovulatory oocyte aging *in vitro*.** (**A**) Distribution of mitochondria with high membrane potential (red) and low membrane potential (green) in three groups of oocytes. (**B**) The fluorescence intensity of ratio of red/green fluorescence analysis for each oocyte was conducted using Image J software. Significant difference between the Aged and 0.1mM Epitalon-treated groups was observed (p < 0.01). (**C**) mtDNA copy numbers of MII oocytes in all three groups were revealed by real-time polymerase chain reaction analysis. Data from more than 30 oocytes were analyzed for each group. Significant difference between the Aged and 0.1mM Epitalon-treated groups was observed (p < 0.01). Data are expressed as mean ± SEM of at least three independent experiments. Scale bar: 100 μm.

Normal mitochondrial membrane potential (MMP) is a prerequisite for mitochondria to carry out respiratory activity, which is necessary for the maintenance of mitochondrial function. In order to detect the effect of Epitalon on the membrane potential of aging oocytes, JC-1 was used to analyze the membrane potential, and the ratio of red/green fluorescence of oocytes was evaluated to reflect mitochondrial function in control, aging, and 0.1mM Epitalon groups. Confocal microscopy showed that active mitochondria were mainly distributed in the peripheral region while low-activity mitochondria were mainly distributed in the central region of the ooplasm (Figure 4A). Meanwhile, the oocyte groups treated with 0.1mM Epitalon showed a higher MMP at 24h of *in vitro* culture as compared to aging oocytes (Figure 4B). In addition, mtDNA copy number at 24h was markedly higher in Epitalon-treated oocytes than in oocytes without Epitalon treatment (Figure 4C), demonstrating that Epitalon has a positive effect on mitochondria to some extent.

### Epitalon alleviates DNA damage and apoptosis of aging oocytes

**Figure 5:**
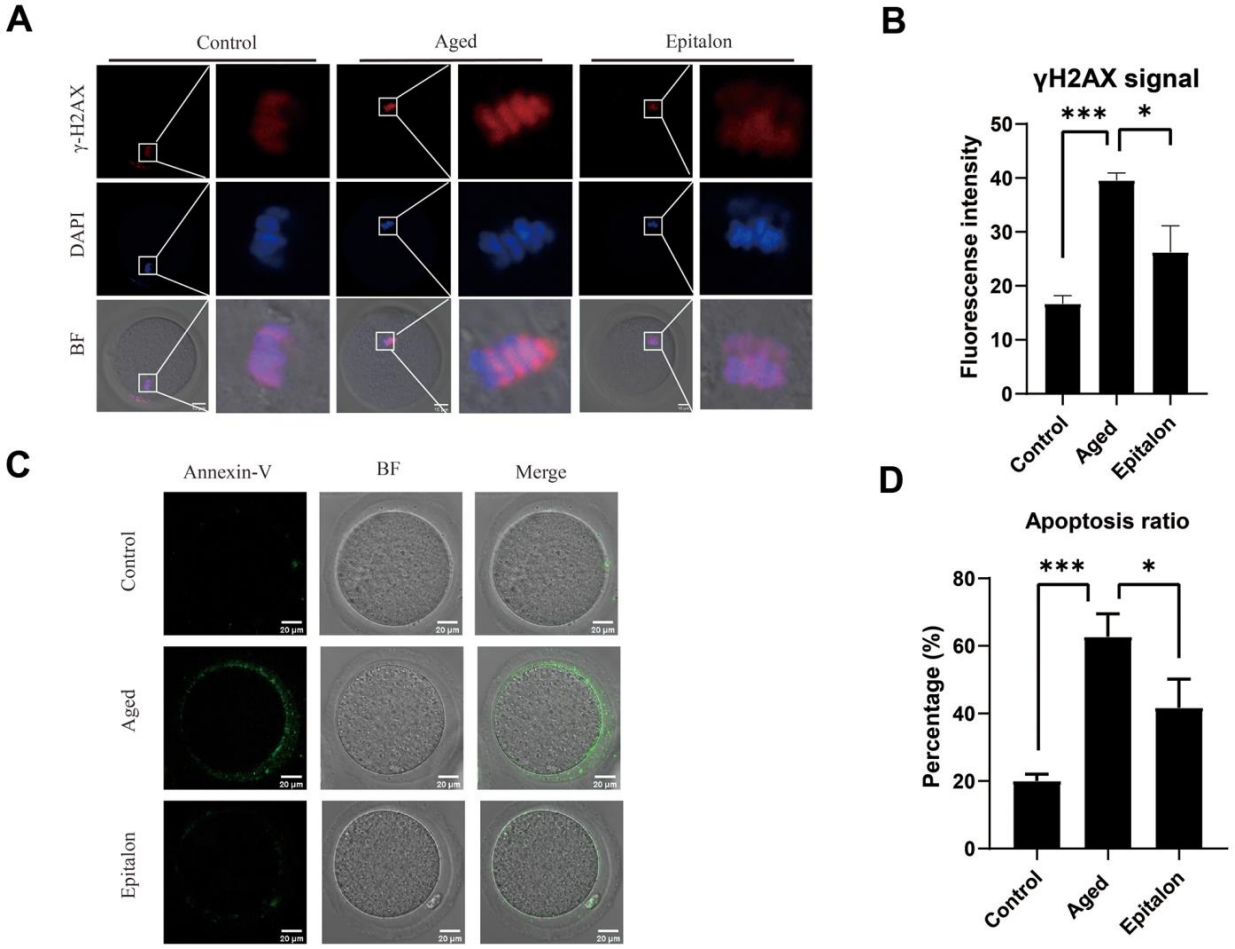
**Effect of Epitalon on early apoptosis in post-ovulatory aging oocytes.** (**A**) Representative images of DNA damage in the Control, Aged, and Epitalon-treated oocytes. Scale bar: 10 μm (**B**) Fluorescence intensity of γ-H2AX signals was measured in the Control, Aged, and Epitalon-treated oocytes. (**C**) Representative images of apoptosis in each group. Oocytes were immunostained with Annexin-V-FITC. (**D**) The rates of early apoptosis were recorded in the Control, Aged, and Epitalon-treated oocytes. Data from more than 30 oocytes were analyzed for each group. Significant difference between the Aged and 0.1mM Epitalon-treated groups was observed (p < 0.05). Data are expressed as mean ± SEM of at least three independent experiments. Scale bar: 20 μm.

Since aging adversely increases oxidative stress and DNA damage, thus accelerating apoptosis of mouse oocytes, we assessed the degree of DNA damage in the aged group and the Epitalon treatment group. As shown (Figure 5A), the fluorescence intensity of γH2AX signals, an indicator of DNA damage, was considerably increased in aged oocytes in comparison with fresh controls. Notably, addition of Epitalon during aging culture was able to alleviate DNA damage (Figure 5B).

Furthermore, mitochondria are involved in the regulation of cell apoptosis [26]. When cells are aging or damaged, the mitochondrial membrane potential decreases, inducing the expression of apoptotic factors such as Cyt-c, which leads to the occurrence of apoptotic processes. Because the distribution and function of mitochondria of ovulatory oocytes were abnormal after 24h of *in vitro* culture, we hypothesized that age-induced mitochondrial abnormalities would accelerate apoptosis. We detected early cell apoptosis by Annexin-V binding to phosphatidylserine, which is flipped from the inside of the lipid membrane to the outside during cell apoptosis. The green fluorescent signal was detected faintly in control oocytes, but it was easily observed in the cell membrane of the aging oocytes (Figure 5C). Moreover, the rate of apoptotic cells was significantly higher in the aging group than in the control group, but it was decreased in the Epitalon-supplemented group (Figure 5D), suggesting that the addition of an appropriate concentration of Epitalon *in vitro* could reduce the degree of early apoptosis of oocytes.

## DISCUSSION

It is not rare for humans and other mammals to have miscarriages or abnormal embryos due to oocyte aging related to maternal age [27]. In human ART, fertilization of fresh oocytes with fresh spermatozoa is important for embryo development. However, asynchrony between oocytes and spermatozoa, especially when aged oocytes are fertilized, will affect the rates of fertilization and embryo development, thus reliable methods are needed to control oocyte aging to benefit modern ART [28]. Seeking agents that retard the oocyte aging process is important. In this study, we investigated whether Epitalon could delay the aging of ovulatory oocytes *in vitro*.

Some antioxidants including melatonin and Epithalamion are peptides secreted by the pineal gland. Epithalamion is a low molecular weight preparation obtained from the pineal gland [29]. Epitalon was designed to restore reproductive function in older rats by increasing testosterone levels in the male blood and restoring the female estrus cycle [30]. Melatonin has also been shown to maintain mitochondrial functions during oocyte aging *in vitro* [31]. Based on these biological effects of peptide bioregulators, we hypothesized that Epitalon can reduce the oxidative stress of aging oocytes and improve the quality of oocytes. The results showed that treatment with 0.1mM Epitalon during 24 h of oocyte aging significantly decreased the level of ROS by maintaining function of mitochondria and thus inhibiting early apoptosis of oocytes.

Oxidative stress occurs in post-aging oocytes, where ROS production increases with a gradual decrease in antioxidant protection [32]. Studies have found that Epitalon restored melatonin formation in the pineal body of older monkeys, increased superoxide dismutase (SOD) activity, and reduced the amount of LPO products, diene conjugates, and ROS [23, 33, 34]. Previous studies have shown that the ROS content in oocytes subjected to aging *in vitro* is time-dependent [35]. The accumulated ROS content during oocytes aging *in vitro* for 24 hours can be easily detected. In order to detect the optimal antioxidant effect of Epitalon in the culture medium, we divided the Epitalon groups into four concentrations of 0.05mM, 0.1mM, 1mM, and 2mM. We found that in the 0.1mM Epitalon group, ROS content was significantly lower, showing that proper concentration of Epitalon protects oocytes against oxidative stress (32.8±5.8 vs. 4.4±1.0, P<0.001). Meanwhile, we detected the fragmentation rate of aged oocytes and the fragmentation rate of oocytes after parthenogenetic activation at the same concentration gradients. We found that oocytes cultured in the medium containing 0.1mM could maintain the normal morphology after *in vitro* aging culture (13.27±0.82% vs. 5.83±0.42%, P=0.0013) and had less cytoplasmic fragmentation after parthenogenetic activation (non-fragmentation rate 59.00±0.58% vs. 31.00±5.13%, P=0.0056). Subsequently, to test whether the quality of oocytes is improved at this concentration, the morphology of spindle and the distribution of CGs in oocytes were statistically analyzed at 6h, 12h, and 24h *in vitro*. We found that the percentages of oocytes with the disrupted spindle morphology and cortical granule free-domain (CGFD) increased during aging compared to fresh oocytes. However, the percentage of abnormal morphology of spindle was decreased at 24h in Epitalon-treated oocytes compared to untreated oocytes (normal spindle rate 69.00±5.34% vs. 48.00±4.16%, P=0.016). In addition, the distribution pattern of CGs was also improved by Epitalon supplementation. All these results suggest a protective role of Epitalon against oocyte aging.

Functional mitochondria are the primary source of ATP production within both oocytes and early embryos [36]. Therefore, it can be considered that the functional status of mitochondria is an important indicator of oocyte cytoplasmic quality. The changes in the functional status mitochondria, as well as the changes of mtDNA copy number during oocyte development, will directly affect the quality of oocytes, and their subsequent embryo development [11]. Mature MII oocytes contain 100,000-200,000 mtDNA copy numbers, which is positively correlated with fertilization and embryonic developmental potential [37]. In addition, the mitochondrial membrane potential (Ψm), which is the polarity of mitochondria, is generated by the activity of mitochondria. There is a proton pump in the mitochondrial intima. The electron transfer in the mitochondrial respiration movement is a process of proton movement. Proton transmembrane transport causes the accumulation of a large number of protons in the mitochondrial membrane space, forming a proton gradient, namely the transmembrane potential across the mitochondrial intima [38]. Normal mitochondrial membrane potential is very important for oocyte quality, and the decrease of mitochondrial membrane potential in oocytes can cause abnormal embryo development [39]. We found that adding a proper concentration of Epitalon during *in vitro* culture could not only maintain normal mtDNA copy numbers (Control, 169821.3±19046.7; aging, 95328.5±1399.5; 0.1mM, 127317.5±22078.1), but also improve mitochondrial functions, as indicated by mitochondrial membrane potential (control, 1.8±0.5; aging, 1.2±0.2; 0.1mM, 1.7±0.2).

Apoptosis is caused by oxidative stress and mitochondrial function damage during aging. ROS can be a signaling molecule to cause DNA damage and apoptosis [40]. We thus measured the signal of γH2AX in nucleus and the extent of phosphatidylserine ectopion in oocytes and showed that supplementing the appropriate concentration of Epitalon during *in vitro* culture can reduce the proportion of early apoptosis in oocytes cultured *in vitro* (39.56±0.78 vs. 26.23±2.83, P<0.001;62.67±3.93 vs. 41.67±4.91, P<0.001).

In conclusion, our study demonstrates that an appropriate concentration of Epitalon can protect oocytes against post-ovulatory aging due to reduced oxidative stress. This finding may be beneficial to subsequent rescue of fertilization using ICSI. Whether Epitalon can improve the quality of oocytes and increase the chance of conception of non-reproductive age women needs further study.

## MATERIALS AND METHODS

### Oocyte collection

All 8-week-old ICR mice were purchased from SPF (Beijing) Biotechnology Co., Ltd. and were kept following policies promulgated by the Ethics Committee of the Institute of Zoology, Chinese Academy of Sciences. To induce superovulation, female mice received intraperitoneal injection of 10 IU PMSG followed 48 h later by 10 IU hCG. The superovulated mice were killed 12-14h after hCG injection and the cumulus cells were removed by pipetting the M2 medium, containing 0.3% hyaluronidase. Only denuded MII oocytes were used for the experiments. They were placed in M2 medium under liquid paraffin oil at 37° C in an atmosphere of 5% CO_2_ in air.

### Oocyte treatment

Collected MII oocytes were washed at least three times and immediately cultured in M2 medium (Cat #M7167; Sigma Aldrich) supplemented without or with different concentrations of Epitalon (Cat #S HY-P1149-5mg). Fresh oocytes, which were collected after 12–14 h of hCG injection without any culture or treatment, were used as a control in our study.

### Detection of intracellular ROS level

ROS levels of three groups of MII oocytes were detected by employing a previously reported method in which ROS Assay Kit was used [41]. The MII oocytes were incubated in M2 medium with 10 mM carboxy-H2DCF diacetate (Cat #S0033; Beyotime, China) at 37° C for 25 min, and washed in M2 medium for at least two times. They have then analyzed at 488 nm (green fluorescence) wavelength by using a confocal laser-scanning microscope (Zeiss LSM 880).

### Time-lapse live imaging experiments

After microinjecting MAP4-eGFP mRNA and H2B-mCherry mRNA, oocytes were incubated for 1h in M2 medium. Microtubule and chromosome dynamics were filmed on a Perkin Elmer precisely Ultra VIEW VOX Confocal Imaging System. Oocytes were exposed once an hour for 10h, and then exposed every 30 min for an extra 7h. Confocal images were acquired with a 20x objective on a spinning disk confocal microscope.

### Immunofluorescence microscopy

Oocytes were fixed in 4% paraformaldehyde in PBS buffer for 30 minutes at room temperature. After being permeabilized with 0.5% Triton X-100 for 20 minutes, they were then blocked in 1% BSA-supplemented PBS for 1 hour at room temperature. For single staining of α-tubulin, oocytes were incubated overnight at 4° C with 1:800 anti-α-tubulin-FITC antibodies (Cat #2125, Beverly, MA). Briefly, oocytes were treated with Tyrode’s solution for 1 min to remove the zona pellucida. Oocytes without zonae pellucidae were fixed with 4% paraformaldehyde in PBS buffer for 30 min at room temperature. After being permeabilized with 0.1% Triton X-100 for 5 minutes, they were then blocked in 1% BSA-supplemented PBS for 1 hour at room temperature, and then cultured with 1:200 lens culinaris (LCA)-FITC for 1 hour at room temperature. Oocytes were washed three times (5 min each time) in PBS buffer. DNA was stained in the final incubation step for 15 min with DAPI. Finally, oocytes were mounted on glass slides and viewed under a confocal laser scanning microscope (Zeiss LSM 880).

### Parthenogenetic activation

After maturation culture, approximately 30 oocytes with the first polar body were collected and treated in preequilibrated Ca^2+^-free (CZB) medium that contained 10 mM SrCl_2_ (A500908; Sangon Biotech, Shanghai, China) for 4–6 hr at 37° C. The presence of pronucleus was regarded as successful parthenogenetic embryos.

### Measurement of mitochondrial membrane potential (MMP)

JC-1, a sensitive cationic carbocyanine fluorescence dye that accumulates in the mitochondria, was applied to measure mitochondrial membrane potential (MMP) of oocytes. Three groups of oocytes were cultured in M2 culture medium with JC-1 (Cat #C2005; Beyotime, China) for 25 min, avoiding direct light. After washing twice with M2 medium, the oocytes were analyzed immediately at 488 nm (for green fluorescence) and 561 nm (red fluorescence) by using a confocal laser-scanning microscope with identical magnification and gain settings throughout the experiments. JC-1 emits red fluorescence in oocytes with highly energized mitochondrial potential (aggregated dye), while depolarized mitochondrial potential emits green fluorescence (monomer dye). A software Image J was used to analyze fluorescence intensity and calculate the mean fluorescence intensity ratio of red to green.

### Mitochondrial DNA content determination by quantitative real-time polymerase chain reaction

Real-time polymerase chain reaction (PCR) was performed to determine the total amount of mtDNA copy number of every single oocyte in all study groups. Briefly, a single oocyte was loaded in a PCR tube with 10 μl lysis buffer (proteinase K added) and incubated at 55° C for 2 hours and 95° C for 10 min, and then the sample was used directly for qPCR analysis. Mouse mtDNA copy number specific primers were used: B6 forward, AACCTGGCACTGAGTCACCA, and B6 reverse, GGGTCTGAGTGTATATATCATGAAGAGAAT. To obtain the standard curve, PCR products amplified with B6 forward and B6 reverse primers were ligated into T-vector. Seven 10-fold serial dilutions of purified plasmid standard DNA were used to generate the standard curve. The cycling conditions included an initial phase of 10 min at 95° C and 40 cycles of 15 s at 95° C and 1 min at 60° C. The melting temperature was 76.5° C. Linear regression analysis of all standard curves for samples with copy numbers between 10^2^ and 10^8^ showed a correlation coefficient higher than 0.98. All measurements were performed in triplicates.

### Annexin-V staining of oocytes

Live oocytes were incubated with Annexin-V-FITC (1:9) (Cat #C1062S; Beyotime, China) in binding buffer for 25 min at room temperature, then the fluorescent signals were examined with a confocal laser scanning microscope (Zeiss LSM 880).

### Statistical analysis

For each experiment, at least three replicates were performed. GraphPad Prism 8.02 (GraphPad Software) was employed to perform statistical analysis. The data were expressed as mean ± SEM and analyzed by one-way ANOVA, statistical significance was ascribed to p< 0.05.
